# Genetic Architecture and Biological Mechanisms of Adolescent Idiopathic Scoliosis: From Susceptibility Loci to Functional Pathways

**DOI:** 10.3390/jcm15207993

**Published:** 2026-10-15

**Authors:** Alexandru Ulici, Alexandru Ciobanu, Alexandru Herdea

**Affiliations:** 1 11th Department of Pediatric Orthopedics, Carol Davila University of Medicine and Pharmacy, Bd. Eroii Sanitari Nr. 8, 050474 Bucharest, Romania; alexandru.ulici@umfcd.ro (A.U.); alexandru.herdea@umfcd.ro (A.H.); 2 Pediatric Orthopedics Department, Grigore Alexandrescu Children’s Emergency Hospital, 011743 Bucharest, Romania

**Keywords:** adolescent idiopathic scoliosis, genetics, genetic susceptibility, genome-wide association study, rare variants, extracellular matrix, biological mechanisms, pathogenesis

## Abstract

Adolescent idiopathic scoliosis (AIS) is a complex three-dimensional spinal deformity with a substantial but incompletely understood genetic component. Advances in genomic research have shifted the genetic landscape of AIS from early candidate-gene associations toward reproducible genome-wide susceptibility loci, rare variants, and functionally relevant regulatory mechanisms. This review synthesizes current evidence across successive levels of AIS genetic investigation—from candidate-gene studies and genome-wide association studies to rare-variant analyses and functional genomics—and integrates these findings according to the biological pathways through which inherited variation may contribute to disease development. Rather than cataloguing individual susceptibility genes, the review specifically examines how genetic findings of different levels of evidence converge on interconnected developmental, cartilage, extracellular-matrix, hormonal/metabolic, and growth-related mechanisms. Genome-wide association studies have consistently implicated loci including *LBX1*, *GPR126/ADGRG6*, *PAX1*, and *SOX6*, supporting the involvement of developmental and musculoskeletal pathways. Rare-variant analyses and functional studies have further implicated extracellular-matrix pathways, including evidence involving *COL11A1* and *COL11A2*. Collectively, current genetic evidence converges on interconnected processes involving neural and somitic development, chondrogenesis, extracellular-matrix organization and remodeling, hormonal and metabolic regulation, and skeletal growth. Functional evidence involving *PAX1*, *GPR126/ADGRG6*, and *COL11A1* provides emerging mechanistic links between genetic variation and spinal development, although the biological consequences of many susceptibility loci remain unresolved. Current evidence supports AIS as a complex polygenic developmental disorder in which common variants, rare variants, and regulatory mechanisms collectively contribute to disease susceptibility. Future research should prioritize functional validation, multi-omics integration, gene–environment interactions, and large multi-ethnic cohorts to advance from genetic association toward a more comprehensive biological understanding of AIS pathogenesis.

## 1. Introduction

Adolescent idiopathic scoliosis (AIS) is the most common spinal deformity affecting children and adolescents and is defined as a three-dimensional deformity of the spine with a Cobb angle of at least 10° in the absence of an identifiable underlying cause. By definition, AIS develops between the age of 10 years and skeletal maturity and affects approximately 2–3% of the adolescent population worldwide [1]. Although most spinal curves remain mild, a proportion of patients develop clinically significant deformity during periods of rapid skeletal growth. Females are affected more frequently than males and are considerably more likely to develop curves requiring conservative or surgical treatment [1]. In patients with more severe deformity requiring surgery, AIS may also have important implications for health-related quality of life and self-image, emphasizing the broader clinical impact of disease severity [2]. In severe AIS, management may require complex surgical strategies, with preoperative halo-gravity traction representing a potential adjunct before posterior spinal fusion in selected patients with severe curves [3].

Despite decades of research, the etiology of AIS remains incompletely understood and is currently considered multifactorial, involving complex interactions between genetic susceptibility, biomechanical factors, hormonal regulation, neuromuscular development, connective-tissue biology, and environmental influences. Familial aggregation, increased concordance among monozygotic twins compared with dizygotic twins, and disease recurrence among first-degree relatives provide strong evidence for an important hereditary component [4,5,6]. However, incomplete concordance even among genetically identical individuals indicates that genetic predisposition alone is insufficient to explain disease development and supports a complex interaction between inherited susceptibility and other biological or environmental factors [4,5,6].

Over the past two decades, advances in genomic research have substantially changed the understanding of the genetic basis of AIS. Early candidate-gene studies primarily investigated genes involved in skeletal growth, hormonal signaling, melatonin metabolism, and connective-tissue organization [6]. The introduction of genome-wide association studies (GWASs) subsequently enabled the identification of reproducible susceptibility loci, including *LBX1*, *GPR126 (ADGRG6)*, *PAX1*, *SOX6*, and several additional loci implicated in musculoskeletal development [7,8,9,10,11,12]. More recently, rare-variant analyses and functional genomic studies have expanded this genetic landscape by implicating extracellular-matrix genes such as *COL11A1* and *COL11A2* and by demonstrating that regulatory variation may alter gene expression during spinal and skeletal development [10,13,14]. These findings increasingly support the concept that AIS is not attributable to a single causative gene but rather reflects a complex polygenic architecture involving common variants, rare variants, and alterations in gene regulation.

Importantly, the biological significance of AIS-associated variants extends beyond their statistical association with disease susceptibility. Genetic findings increasingly converge on interconnected pathways involved in neural and somitic development, chondrogenesis, extracellular-matrix organization and remodeling, hormonal and metabolic regulation, and skeletal growth. Functional studies involving loci such as *PAX1*, *GPR126*, and *COL11A1* have begun to provide mechanistic links between genetic variation and biological processes relevant to spinal development [8,10,14]. Nevertheless, the functional consequences of many susceptibility loci remain incompletely characterized, and substantial genetic heterogeneity exists across populations.

This review aims to synthesize the current evidence on the genetic architecture of AIS susceptibility and to integrate genetic discoveries with the biological mechanisms through which they may contribute to disease development. Particular emphasis is placed on the transition from early candidate-gene studies to genome-wide association studies, rare-variant analyses, and functional investigations, and on the convergence of these findings across developmental, cartilage, extracellular-matrix, hormonal, metabolic, and growth-related pathways. By connecting genetic associations of different levels of evidence with their potential biological consequences, this review seeks to provide an integrated pathway-based framework for understanding how inherited variation may contribute to AIS pathogenesis, while distinguishing well-replicated genetic findings from emerging mechanistic hypotheses and identifying the principal gaps that should guide future genetic and functional research. Previous reviews have addressed specific components of AIS genetics, including genetic epidemiology, candidate susceptibility loci, extracellular-matrix biology, epigenetic regulation, and genetic or epigenetic factors associated with curve progression. The present review adopts a complementary mechanistic perspective by organizing evidence across successive levels of genetic investigation—from candidate-gene associations and replicated genome-wide susceptibility loci to rare variants and functional studies—and then examining how these findings converge on shared biological pathways. Particular emphasis is placed on distinguishing statistical genetic association from functional evidence and on identifying mechanistic connections between developmental regulation, chondrogenesis, extracellular-matrix organization and remodeling, hormonal and metabolic signaling, and skeletal growth. This framework is intended to move beyond a catalogue of AIS-associated genes toward an integrated interpretation of how multiple forms of inherited variation may collectively contribute to disease susceptibility [6,15,16,17].

## 2. Literature Search Approach

This article was designed as a narrative review aimed at integrating genetic findings in adolescent idiopathic scoliosis (AIS) with their potential biological and mechanistic implications. The literature was identified through searches of PubMed, Web of Science, and ScienceDirect, with particular emphasis on studies published during the last 10 years (2016–2026), while earlier landmark studies were retained when necessary to describe the historical development and replication of major AIS susceptibility loci. The literature search was last updated in October 2026. Search terms included combinations of “adolescent idiopathic scoliosis” or “AIS” with “genetics”, “genetic susceptibility”, “candidate gene”, “polymorphism”, “single-nucleotide polymorphism” or “SNP”, “genome-wide association study” or “GWAS”, “rare variants”, “functional genomics”, “gene regulation”, “epigenetics”, “extracellular matrix”, “cartilage”, “chondrogenesis”, “skeletal growth”, and “biological mechanisms”. Priority was given to original genetic studies, genome-wide investigations, replication studies, rare-variant analyses, and studies providing functional or mechanistic evidence relevant to AIS susceptibility. Additional relevant publications were identified from the reference lists of selected articles.

Because the objective was to provide a narrative and mechanistic synthesis rather than an exhaustive systematic review, study selection was based on relevance to the genetic architecture and biological pathways discussed in the manuscript rather than on a formal systematic-review selection protocol. Accordingly, a PRISMA flow diagram was not generated. The studies presented in Table 1 were selected as representative examples illustrating key stages in the evolution of AIS genetic research, including candidate-gene approaches, genome-wide association studies, rare-variant analyses, and functional investigations. Particular emphasis was placed on replicated susceptibility loci, findings that substantially expanded the genetic architecture of AIS, and studies providing functional evidence linking genetic variation to biological mechanisms.

As a narrative review, this approach has inherent limitations related to selective literature coverage. Although priority was given to replicated genetic findings, recent genomic studies, and investigations providing functional or mechanistic evidence, the review was not designed to identify or quantitatively assess every published AIS association study. Consequently, some relevant studies may not have been included, and the studies summarized in the text and tables should be interpreted as representative rather than exhaustive.

## 3. Genetic Architecture of AIS Susceptibility

### 3.1. Early Candidate Gene Studies

Early genetic investigations of adolescent idiopathic scoliosis were largely based on candidate-gene approaches, in which genes were selected according to their presumed involvement in biological processes potentially relevant to AIS pathogenesis. These studies primarily focused on pathways related to skeletal growth, hormonal signaling, bone metabolism, connective-tissue organization, and melatonin-related mechanisms. Although candidate-gene studies provided some of the earliest evidence linking specific biological pathways to AIS, many reported associations were inconsistent across populations or were not reproduced in subsequent studies, highlighting the genetic and phenotypic heterogeneity of the disorder [6].

One of the early candidate-gene studies was conducted by Qiu et al. (2007) [18], who investigated whether polymorphisms in the growth hormone receptor gene (GHR) were associated with AIS susceptibility or clinical phenotype. The study included 510 female patients with AIS and 363 healthy controls and evaluated five *GHR* polymorphisms. No significant differences in genotype or allele frequencies were identified between patients and controls, and no association was observed between GHR genotype and curve severity. These findings suggested that *GHR* is unlikely to represent a major independent susceptibility or disease-modifying gene in AIS. Nevertheless, the study was relevant in directing attention toward growth-regulatory pathways because of the close relationship between pubertal skeletal growth and the clinical manifestation of AIS.

Growth-related pathways were further investigated by Moon et al. (2013) [19], who evaluated genetic polymorphisms in a Korean AIS population. Associations involving *IGF1* and *LAPTM4B* were reported in relation to AIS phenotype, including susceptibility and curve severity. *IGF1* is of particular biological interest because of its central role in longitudinal skeletal growth and growth-plate activity during adolescence. These findings supported the hypothesis that genetic variation affecting growth-regulatory pathways may contribute to interindividual differences in AIS phenotype. However, as with many early candidate-gene studies, population specificity and limited independent replication restrict the generalizability of these associations.

Metabolic and hormonal pathways have also been investigated as potential contributors to AIS susceptibility. Araujo Junior et al. (2024) [20] examined *LEP* rs2167270 and *LEPR* rs2767485 in 189 girls with AIS and 240 controls. *LEPR* rs2767485 was associated with AIS susceptibility, whereas *LEP* rs2167270 was associated with lower body mass index among girls with AIS. Given the role of leptin signaling in energy metabolism, skeletal growth, and bone homeostasis, these findings suggest a possible link between metabolic regulation and the development of AIS. Nevertheless, the functional consequences of AIS-associated *LEPR* variation remain insufficiently characterized, and further replication across independent populations is required.

Taken together, candidate-gene studies contributed to the early understanding of the biological complexity of AIS by implicating pathways related to growth, hormonal regulation, metabolism, and skeletal development. However, inconsistent replication, relatively small or ethnically homogeneous cohorts, and the investigation of a limited number of preselected variants substantially restricted their ability to define the genetic architecture of the disease. These limitations ultimately encouraged the transition toward hypothesis-free genome-wide approaches capable of identifying susceptibility loci without prior assumptions regarding the biological pathways involved [6,7,11].

### 3.2. Genome-Wide Association Studies

The introduction of genome-wide association studies represented a major transition in AIS genetics. In contrast to candidate-gene approaches, GWASs enable the systematic evaluation of large numbers of common genetic variants across the genome without requiring prior assumptions regarding the biological pathways involved. This strategy led to the identification of several reproducible susceptibility loci and substantially strengthened the evidence supporting a complex polygenic architecture for AIS [7,8,11,12].

A major breakthrough was reported by Takahashi et al. (2011) [7], who performed a genome-wide association study in Japanese cohorts and identified common variants near *LBX1* associated with AIS susceptibility. The association was subsequently replicated in independent populations, establishing the *LBX1* region as one of the most consistently supported genetic loci in AIS. *LBX1* encodes a transcription factor involved in developmental and neuromuscular processes, providing an early indication that disturbances in developmental gene regulation may contribute to AIS pathogenesis.

Kou et al. (2013) [8] subsequently identified genetic variants in *GPR126*, currently designated *ADGRG6*, as being associated with AIS. This finding expanded the genetic landscape beyond LBX1 and was particularly important because *GPR126* is involved in developmental processes relevant to cartilage and skeletal biology. Functional investigations provided additional biological support for the association, strengthening the link between genetic susceptibility and pathways involved in spinal and cartilage development.

Further expansion of the AIS susceptibility landscape was provided by Zhu et al. (2015) [9], who performed a GWAS in Chinese girls and identified additional susceptibility loci, including regions involving *AJAP1*, *PAX3/EPHA4*, *BCL2*, and *LBX1-AS1*. These findings demonstrated that AIS susceptibility is distributed across multiple genomic regions and reinforced the concept that several developmental pathways may contribute simultaneously to disease risk.

In the same year, Sharma et al. (2015) [10] provided particularly important evidence connecting genetic association with functional regulation. Using a two-stage genome-wide approach involving 3102 participants, the authors identified a female-specific susceptibility locus associated with *PAX1*. The associated variants were located within a non-coding enhancer involved in the regulation of *PAX1* expression during vertebral development. Functional assays demonstrated that risk-associated variants altered enhancer activity, providing evidence that AIS susceptibility may result not only from protein-coding mutations but also from regulatory variants capable of modifying developmental gene expression. The sex-specific nature of the association was also particularly relevant given the marked female predominance observed in clinically significant AIS.

Functional evidence has also been reported for *BNC2*, another AIS susceptibility locus. Xu et al. [21] investigated the functional variant rs10738445 in a Chinese population and confirmed its association with AIS in a two-stage case–control analysis. The risk allele was associated with increased AIS susceptibility, while *BNC2* expression was higher in patients with AIS and correlated with curve severity. These findings provided evidence linking a susceptibility-associated regulatory variant to altered gene expression and clinical phenotype, although further functional validation and replication across diverse populations remain necessary [21].

Khanshour et al. (2018) [11] extended these observations through a genome-wide meta-analysis followed by replication across multiple ethnic populations. The study identified additional susceptibility loci, including *SOX6*, and demonstrated enrichment of genetic signals in biological pathways related to cartilage and connective-tissue development. *SOX6* is involved in chondrocyte differentiation and cartilage formation, while its convergence with other developmental genes further supported the hypothesis that disturbances in vertebral and skeletal development contribute to AIS susceptibility. Importantly, the multi-ethnic design represented an advance over many earlier studies conducted within single populations and highlighted both shared and population-dependent components of AIS genetic risk.

The polygenic nature of AIS was further reinforced by Kou et al. (2019) [12], who conducted a large GWAS in a Japanese population and identified 14 previously unreported susceptibility loci. The substantial expansion in the number of associated genomic regions demonstrated that AIS susceptibility cannot be explained by a small number of major-effect genes. Instead, the accumulating GWAS evidence supports a genetic architecture in which numerous common variants, each generally contributing a relatively modest effect, collectively influence disease susceptibility.

Overall, GWASs transformed the understanding of AIS genetics from a candidate-gene model toward a complex polygenic framework. Reproducible loci involving *LBX1*, *GPR126/ADGRG6*, *PAX1*, *SOX6*, and additional developmental genes converge on biological processes involving neural and somitic development, cartilage formation, connective-tissue biology, and spinal morphogenesis. Nevertheless, GWAS associations primarily identify genomic regions statistically associated with disease and do not by themselves establish causality. Functional characterization of associated variants therefore remains essential for determining how these loci contribute to the biological development of AIS.

### 3.3. Rare Variants, Extracellular-Matrix Genes, and Whole-Genome Sequencing

Although GWASs have successfully identified numerous common susceptibility variants, the effect sizes of individual loci are generally modest and explain only part of the genetic contribution to AIS. This limitation has stimulated increasing interest in rare genetic variants, which may exert larger biological effects and provide additional insight into disease mechanisms that are not captured by common-variant association studies [11,12,13].

Haller et al. (2016) [13] investigated the contribution of rare variants to AIS and identified an increased polygenic burden of rare variants across genes involved in extracellular-matrix organization. Rather than implicating a single causative mutation, the study suggested that the cumulative effect of multiple rare variants affecting structurally and functionally related genes may contribute to AIS susceptibility. *COL11A2* was among the genes implicated, supporting a potential role for altered collagen organization and extracellular-matrix integrity in the pathogenesis of the disorder.

These findings are biologically plausible because the extracellular matrix provides structural support to vertebral cartilage, intervertebral tissues, ligaments, and other components of the developing spine. Alterations affecting collagen fibril organization or matrix integrity could potentially modify the biomechanical properties of spinal tissues during periods of rapid growth. The rare-variant model therefore complements common-variant GWAS findings by suggesting that AIS genetic architecture may include both relatively frequent alleles with modest effects and less common variants with potentially greater functional consequences [13,14].

Yu et al. (2024) [14] provided genetic and functional evidence implicating *COL11A1* in AIS. Importantly, the principal association involved rs3753841 (p.Pro1335Leu), a common missense variant, and should therefore be distinguished from rare-variant findings involving extracellular-matrix genes. The study further investigated the biological consequences of *COL11A1* dysregulation and identified a regulatory relationship involving *PAX1*, *COL11A1*, and downstream extracellular-matrix remodeling. These findings provide a mechanistic connection between a common *COL11A1*-associated variant, altered gene regulation, and extracellular-matrix biology relevant to AIS.

More recently, Tuncay et al. (2025) [22] applied whole-genome sequencing to 119 individuals with AIS from 103 families, extending genetic investigation beyond predefined candidate loci and common-variant association studies. The analysis identified coding, non-coding, and structural candidate variants, including variants in previously implicated AIS genes such as *COL11A2* and *FBN1*. Gene-set analyses demonstrated convergence across several biological processes, including extracellular-matrix organization, skeletal-muscle contraction, and regulation of gene expression. These findings further support a genetically heterogeneous and polygenic model of AIS while illustrating the potential of whole-genome sequencing to identify multiple classes of genetic variation within the same analytical framework [22].

The increasing focus on rare variants and functional genomics represents an important evolution in AIS research. Whereas early candidate-gene studies examined a limited number of biologically plausible polymorphisms and GWASs identified common susceptibility loci through population-level association, rare-variant and functional approaches increasingly seek to determine how genetic variation modifies biological processes relevant to spinal development. This transition is particularly important because statistical association alone cannot establish the functional consequences of a susceptibility locus.

Taken together, current evidence supports a genetic architecture of AIS involving multiple levels of variation, including common susceptibility loci, rare variants, and non-coding regulatory variants. Rather than acting through a single molecular pathway, these genetic factors appear to converge on interconnected biological processes involving developmental regulation, cartilage formation, extracellular-matrix organization, and skeletal growth. This emerging framework provides the basis for interpreting AIS as a complex polygenic developmental disorder and leads directly to the biological mechanisms discussed in the following section.

Across these studies, several areas of convergence can be identified despite substantial methodological and population heterogeneity. Replicated common-variant associations involving loci such as *LBX1*, *ADGRG6*, and *PAX1* consistently implicate developmental regulation, whereas rare-variant and whole-genome sequencing studies increasingly converge on extracellular-matrix organization, cartilage biology, skeletal-muscle function, and regulation of gene expression. Functional investigations of *BNC2*, *PAX1*, and *ADGRG6* further support the concept that at least some AIS susceptibility signals act through altered gene regulation rather than protein-coding disruption alone. However, important discrepancies remain. Many associations are population-specific or incompletely replicated, effect sizes of common susceptibility variants are generally modest, and candidate-gene findings have often been inconsistent across cohorts. Moreover, functional evidence is available for only a subset of associated loci, and experimental phenotypes cannot necessarily be directly extrapolated to human AIS. Major unresolved questions therefore include the identity of the causal variants underlying many GWAS signals, the relative contribution of common versus rare variation, the mechanisms through which genetically distinct loci converge on shared biological pathways, and the extent to which these mechanisms influence disease susceptibility, curve severity, or progression.

Representative studies illustrating the evolution of AIS genetic research from candidate-gene approaches to genome-wide association studies, rare-variant analyses, and functional investigations are summarized in Table 1. Studies were selected for inclusion in the table based on their relevance to the principal genetic approaches and biological pathways discussed in this narrative review, with emphasis on replicated susceptibility loci, studies introducing important genetic findings, and investigations providing functional or mechanistic evidence. The table is intended to illustrate key stages in the development of AIS genetic research rather than to provide an exhaustive catalogue of all published association studies.

## 4. Biological Mechanisms Linking Genetic Susceptibility to AIS

### 4.1. Neural and Somitic Development

The increasing number of genetic variants associated with adolescent idiopathic scoliosis indicates that AIS susceptibility cannot be attributed to a single molecular pathway. Instead, available genetic and functional evidence suggests that multiple biological processes converge during spinal growth and development. Genes implicated in AIS participate in neural and somitic development, chondrogenesis, extracellular matrix organization, hormonal and metabolic signaling, and regulation of skeletal growth [7,8,10,11,13,14,20]. Alterations in these interconnected pathways may influence vertebral development, connective-tissue properties, neuromuscular organization, and the response of the growing spine to mechanical forces, thereby creating a biological predisposition to spinal asymmetry.

Abnormalities in early neural, muscular, and somitic development represent one potential mechanism linking genetic susceptibility to AIS. Among the genes supporting this hypothesis, *LBX1* and *PAX1* are particularly relevant because both encode transcription factors involved in developmental processes affecting structures that contribute to spinal formation and stability [7,10].

*LBX1* is a homeobox transcription factor involved in embryonic development, particularly in the specification and migration of muscle precursor cells and in neural development. Its identification as one of the strongest and most consistently replicated AIS susceptibility loci suggested that disturbances in developmental pathways involving the neuromuscular system may contribute to disease susceptibility [7]. Altered regulation of *LBX1* could potentially influence the development or function of paraspinal musculature and neural pathways involved in postural control. However, despite the robust genetic association between the LBX1 region and AIS, the precise molecular mechanisms through which associated variants contribute to spinal asymmetry remain incompletely understood.

*PAX1* provides a complementary link between genetic susceptibility and axial skeletal development. *PAX1* is a transcription factor involved in somite differentiation and vertebral development. AIS-associated variants identified near *PAX1* are located within non-coding regulatory regions, supporting the hypothesis that altered gene regulation, rather than disruption of the protein-coding sequence itself, may contribute to disease susceptibility. Functional evidence demonstrating altered enhancer activity further suggests that inappropriate regulation of developmental genes during spinal formation may represent an important mechanism in AIS [10].

More recent evidence has strengthened the biological relevance of *PAX1* by connecting it with downstream extracellular matrix pathways, particularly the regulation of *COL11A1*. This relationship suggests that developmental transcriptional regulation may influence not only early vertebral patterning but also the subsequent structural properties of cartilage and extracellular matrix. Thus, *PAX1* may represent an important molecular link between early vertebral development and cartilage and extracellular matrix homeostasis. Together, *LBX1* and *PAX1* support the hypothesis that subtle disturbances in developmental gene regulation may predispose the growing spine to the structural asymmetry characteristic of AIS [7,10,14].

### 4.2. Cartilage Development and Chondrogenesis

Normal vertebral growth depends on tightly regulated chondrogenesis and cartilage maturation. Several AIS-associated genes, including *GPR126/ADGRG6*, *SOX6*, *COL11A1*, and *COL11A2*, are involved in cartilage development or maintenance, suggesting that impaired cartilage biology may represent an important pathway contributing to disease susceptibility [8,11,14,23].

*GPR126/ADGRG6* emerged as an important AIS susceptibility gene following genome-wide association studies and subsequent functional investigations. Experimental evidence has strengthened the biological interpretation of this association. In a mouse model, conditional deletion of *Gpr126* in osteochondroprogenitor cells resulted in postnatal scoliosis without congenital vertebral malformations, together with abnormalities of cartilage and intervertebral-disc development, including impaired annulus fibrosus formation and increased chondrocyte apoptosis. These findings provide functional evidence that disruption of *GPR126/ADGRG6*-dependent cartilage biology can affect postnatal spinal development, supporting a mechanistic connection between the human susceptibility locus and scoliosis-related phenotypes [8,23]. The identification of *SOX6* further strengthened the relationship between AIS and chondrogenesis. *SOX6* is a transcription factor with an important role in chondrocyte differentiation and cartilage formation. Genetic evidence implicating *SOX6* therefore suggests that alterations in transcriptional programs governing cartilage development may affect the structural development of the vertebral column [11]. The convergence of *GPR126* and *SOX6* on cartilage-related biological processes supports the concept that AIS-associated variants located at different genomic loci may influence shared developmental pathways.

Collagen genes provide an additional connection between cartilage biology and AIS. *COL11A1* and *COL11A2* encode components of type XI collagen, which contributes to the organization and structural integrity of cartilage extracellular matrix. Rare-variant analyses have demonstrated an increased burden of variants affecting collagen and extracellular matrix genes, including *COL11A2*, while more recent genetic and functional evidence has implicated *COL11A1* in AIS susceptibility [13,14].

Functional investigation of *COL11A1* has further connected cartilage development with PAX1-mediated gene regulation. Alterations in this pathway may disturb collagen organization and cartilage homeostasis during spinal growth. Taken together, evidence involving *GPR126/ADGRG6*, *SOX6*, *COL11A1*, and *COL11A2* suggests that impaired chondrogenesis and cartilage integrity may create a biological environment in which normal vertebral development becomes more susceptible to asymmetric deformation [8,11,13,14].

### 4.3. Extracellular Matrix Organization and Remodeling

The extracellular matrix provides both structural support and biochemical signals necessary for the normal development and maintenance of spinal tissues [13,14,25]. Genetic alterations affecting ECM components or their regulation may therefore influence the mechanical properties of cartilage, ligaments, intervertebral structures, and other connective tissues involved in spinal stability.

Among the strongest genetic evidence supporting this pathway are *COL11A1* and *COL11A2*. Type XI collagen plays an important role in maintaining collagen fibril organization and the structural integrity of cartilage. Genetic variation affecting these genes may consequently modify extracellular matrix architecture and alter the mechanical properties of spinal tissues. The enrichment of rare variants across multiple collagen and *ECM* genes further suggests that cumulative disruption of matrix components, rather than a defect in a single structural protein, may contribute to AIS susceptibility [13,14].

Other connective-tissue genes, including *FBN1* and *FBN2*, provide additional evidence for the involvement of extracellular matrix biology in AIS. Fibrillins are major components of extracellular microfibrils and contribute to tissue elasticity, structural integrity, and connective-tissue signaling. Rare variants in *FBN1* and *FBN2* have been reported in association with severe AIS [25]. Importantly, these findings concern a severe-disease phenotype and should not be interpreted as demonstrating an increased risk of developing AIS in the general population or as predicting subsequent curve progression. The available evidence remains more limited than that supporting established GWAS susceptibility loci, and further independent replication and longitudinal investigations are required.

Recent genome-wide evidence further supports the distinction between genetic susceptibility to AIS and genetic determinants of curve severity. Dai et al. (2026) [24] conducted a two-stage GWAS involving Han Chinese female patients with AIS, comparing severe curves (>40°) with mild curves (<30°) at skeletal maturity. The discovery cohort included 620 patients, followed by an independent replication cohort of 1180 patients. Two intronic variants showed significant replication: rs2061846 in GAK (OR = 1.32; 95% CI: 1.11–1.57; *p* = 0.001) and rs7330031 in KLF12 (OR = 1.46; 95% CI: 1.16–1.85; *p* = 0.001). Additionally, *KLF12* mRNA expression in paraspinal muscle samples was inversely correlated with Cobb angle (r = −0.451; *p* = 0.027), suggesting a possible relationship between transcriptional regulation, muscle biology, and curve magnitude. However, these results primarily establish associations with curve severity rather than AIS susceptibility or prospectively observed progression. Although both loci were replicated within the study, independent validation across populations and prospective cohorts is required before their potential use in clinical prognostic screening can be established [24].

Extracellular matrix homeostasis also depends on controlled degradation and remodeling. *MMP3*, a matrix metalloproteinase involved in the degradation of extracellular matrix components, represents an important component of this process. Functional evidence linking *PAX1* and *COL11A1* with *MMP3* suggests that AIS-associated genetic variation may affect not only the production and organization of structural matrix proteins but also their turnover and remodeling [14].

Consequently, extracellular matrix abnormalities in AIS may involve an imbalance between matrix synthesis, organization, and degradation. Such alterations could modify the biomechanical properties of spinal tissues during growth and increase their susceptibility to asymmetric deformation. The convergence of collagen genes, fibrillin-related pathways, and matrix-remodeling mechanisms therefore supports extracellular matrix dysregulation as an important component of AIS pathogenesis [13,14,25].

### 4.4. Hormonal and Metabolic Pathways

The marked female predominance of clinically significant AIS and the frequent emergence of spinal deformity during puberty have stimulated considerable interest in hormonal and metabolic mechanisms. In the context of AIS genetics, these factors should not be interpreted as altering the inherited DNA sequence itself. Rather, hormonal and metabolic signals may interact with an underlying genetic susceptibility by modifying gene expression, cellular signaling, skeletal maturation, and tissue responses during periods of rapid growth. Epigenetic regulation represents one plausible molecular interface through which such interactions may occur [26]. Accordingly, estrogen signaling, leptin signaling, vitamin D metabolism, and serotonin–melatonin pathways may be better considered potential modifiers of genetically susceptible biological systems than independent primary genetic causes of AIS. Estrogen signaling has received particular attention because of the marked sex difference observed in AIS. Variants in ESR1 and ESR2 have been investigated in multiple populations, based on the hypothesis that altered estrogen signaling may influence skeletal maturation, bone metabolism, and spinal growth. Although associations with AIS-related phenotypes have been reported, findings have not been consistently replicated across populations. Overall, the available evidence suggests that estrogen-related pathways may contribute to the biological context in which AIS develops, although individual estrogen receptor variants cannot currently be regarded as consistently established major susceptibility loci [26]. Importantly, studies investigating estrogen receptor variants have assessed different clinical outcomes, including AIS susceptibility, curve severity, and, in some cases, curve progression. These outcomes should not be considered interchangeable. In particular, cross-sectional associations with Cobb angle do not establish longitudinal progression, and associations observed among patients with AIS do not necessarily indicate increased susceptibility to developing the disease. Moreover, the inconsistent replication of estrogen receptor associations limits their current utility as clinically validated prognostic biomarkers.

Leptin signaling provides another potential connection between systemic metabolism and skeletal development. Araujo Junior et al. (2024) [20] reported an association between *LEPR* rs2767485 and AIS susceptibility, whereas *LEP* rs2167270 was associated with lower body mass index among girls with AIS. Because leptin participates in energy homeostasis, bone metabolism, and skeletal growth, altered leptin signaling could potentially influence the biological environment in which spinal deformity develops. Nevertheless, the functional consequences of AIS-associated *LEPR* variation remain insufficiently characterized.

The outcomes investigated in this study should be distinguished. The association involving *LEPR* rs2767485 concerned AIS susceptibility, whereas the association involving *LEP* rs2167270 concerned BMI among girls already diagnosed with AIS. Therefore, the BMI finding represents an associated metabolic phenotype and does not independently establish genetic susceptibility to AIS, curve severity, or subsequent curve progression.

Vitamin D-related pathways have also been investigated because of their role in bone mineralization and skeletal development. Genetic variation involving vitamin D signaling has been associated with AIS-related phenotypes in individual studies, although the available evidence remains heterogeneous and population-dependent [27]. Similarly, pathways involving serotonin and melatonin signaling have been proposed as potential contributors to AIS biology. However, their contribution to primary disease susceptibility remains less firmly established than that of replicated developmental loci identified through genome-wide studies [28].

Taken together, current evidence supports a model in which hormonal and metabolic factors interact with, rather than replace, inherited genetic susceptibility. Their contribution may become particularly relevant during puberty, when rapid skeletal growth coincides with major changes in sex hormones, energy metabolism, and bone remodeling. These systemic signals may influence the penetrance or phenotypic expression of susceptibility variants through changes in transcriptional activity, cellular signaling, and potentially epigenetic regulation. This framework may also help explain why genetic susceptibility does not translate uniformly into clinically apparent AIS and why disease expression varies according to sex, developmental stage, and individual biological context. However, direct evidence linking specific hormonal or metabolic exposures to epigenetic regulation of established AIS susceptibility loci remains limited, and this hypothesis requires validation in longitudinal and functional studies. Although genetic and molecular findings provide valuable insights into AIS pathogenesis, their potential clinical predictive value remains insufficiently established. Statistical associations with disease susceptibility, curve severity, or related phenotypes should not be interpreted as validated tools for predicting individual disease onset or future curve progression. Clinical implementation will require independent replication, prospective longitudinal validation, assessment of predictive performance, and demonstration of added value beyond established clinical and radiographic risk factors.

### 4.5. Non-Coding RNA and Epigenetic Regulation in AIS

Beyond protein-coding susceptibility loci, non-coding regulatory mechanisms have emerged as potentially important contributors to AIS pathogenesis and curve progression. Ogura et al. [29] identified a genome-wide significant locus at *MIR4300HG* associated with curve progression in 2543 Japanese patients with AIS (OR = 1.56, 95% CI: 1.35–1.80; *p* = 1.98 × 10^−9^). Functional assays demonstrated that the risk allele of rs35333564 reduced enhancer activity, suggesting a regulatory mechanism involving *MIR4300HG* and its embedded microRNA, *MIR4300*. Subsequent findings in a Chinese population supported an association with curve severity and progression-related phenotypes, but not with AIS susceptibility.

Importantly, progression-related phenotypes in these studies were defined using study-specific criteria involving curve magnitude and skeletal maturity. These associations should not be interpreted as prospectively validated predictions of future curve progression, which would require longitudinal follow-up and independent validation. These findings highlight the importance of distinguishing genetic determinants of disease occurrence from those associated with curve deterioration [29,30].

Other non-coding RNAs, particularly microRNAs, have been investigated as potential regulators of musculoskeletal development and AIS-associated molecular pathways. However, their specific contributions to disease initiation and progression remain incompletely established, and many reported associations require independent functional validation [31].

Epigenetic studies have also identified tissue-specific DNA methylation patterns involving estrogen receptor genes in paraspinal muscles. *ESR1* methylation at tissue-dependent regulatory regions has been associated with gene expression and cross-sectional curve severity, whereas *ESR2* promoter methylation differs between the concave and convex sides of the curvature without a demonstrated association with Cobb angle severity. These observations suggest that epigenetic regulation may contribute to tissue-specific responses during spinal development. Nevertheless, cross-sectional methylation differences cannot establish whether these changes precede scoliosis development or arise as secondary adaptations to spinal deformity [32,33].

Together, these findings expand the genetic framework of AIS beyond conventional protein-coding variants, while emphasizing the need for longitudinal studies, independent replication, and functional investigation in disease-relevant tissues.

### 4.6. Ciliary Dysfunction and Cerebrospinal Fluid Dynamics in AIS

Ciliary dysfunction has emerged as a potential mechanism linking genetic alterations to abnormal spinal morphogenesis. Primary cilia function as sensory organelles involved in mechanotransduction and developmental signaling, whereas motile cilia contribute to cerebrospinal fluid (CSF) circulation. Although these structures have distinct functions, experimental studies suggest that disturbances in ciliary biology may affect the maintenance of spinal alignment.

*POC5*, a centrosomal protein implicated in AIS, provides experimental evidence supporting this hypothesis. Hassan et al. [34] demonstrated that cells expressing the AIS-associated *POC5* p.A429V variant exhibited abnormal subcellular localization, reduced ciliary length, impaired cell-cycle progression, and altered interactions with centrosomal and cytoskeletal proteins. These findings support a potential connection between a human AIS-associated variant and cellular dysfunction, although its contribution to spinal deformity in vivo remains to be established [34].

Complementary evidence comes from zebrafish models. Grimes et al. [35] demonstrated that disruption of motile cilia and cerebrospinal fluid (CSF) flow can induce scoliosis-like spinal curvature, while restoration of ciliary motility after curve onset prevented further progression [35]. Subsequent experimental work demonstrated that the Reissner fiber, an extracellular structure within the CSF, contributes to spinal morphogenesis through urotensin-related signaling in CSF-contacting neurons. Disruption of this pathway was associated with spinal curvature in zebrafish, providing a mechanistic link between CSF-associated signaling, neuromuscular regulation, and axial morphology [36]. These findings support a potential relationship between ciliary function, fluid dynamics, and spinal development, although their direct relevance to human AIS remains to be established.

Nevertheless, mechanisms established in zebrafish should not be assumed to operate identically in human AIS. In particular, the direct relationship between human ciliary gene variants, altered CSF dynamics, and progressive spinal deformity requires further investigation. Ciliary dysfunction therefore represents a biologically plausible but incompletely validated component of AIS pathogenesis.

### 4.7. Growth Regulation

Rapid longitudinal skeletal growth during adolescence coincides with the period in which AIS most commonly becomes clinically apparent [1,18,19]. Consequently, genes regulating skeletal growth have long been considered biologically plausible contributors to disease pathogenesis. *GHR* and *IGF1* are particularly relevant because the growth hormone–insulin-like growth factor axis represents a major regulator of longitudinal bone growth and skeletal maturation.

The growth hormone receptor (GHR) mediates the biological effects of growth hormone and contributes to the regulation of vertebral and long-bone growth. Genetic variation affecting GHR could theoretically modify the response of skeletal tissues to growth hormone and create differences in growth velocity or vertebral development. However, available association studies have not provided consistent evidence supporting common GHR polymorphisms as major determinants of AIS susceptibility [18]. The growth hormone pathway therefore remains biologically relevant, but GHR itself cannot currently be considered an established major AIS susceptibility gene.

*IGF1* acts downstream of growth hormone and has a central role in chondrocyte proliferation, growth-plate activity, and skeletal development. Genetic associations involving *IGF1* have been reported in relation to AIS susceptibility and clinical phenotype [19]. These observations support the possibility that variation in growth-regulatory pathways may modify the expression of spinal deformity during adolescence. Nevertheless, available studies remain relatively limited and require replication in larger independent populations.

Rather than indicating a primary defect of the growth hormone–*IGF1* axis, the available evidence suggests that growth-related pathways may modify the expression of an underlying genetic predisposition. During the adolescent growth spurt, subtle alterations in vertebral growth, cartilage biology, extracellular matrix properties, or neuromuscular organization may become amplified as the spine rapidly increases in size. Growth regulation may therefore interact with developmental, structural, hormonal, and metabolic mechanisms to contribute to the emergence of spinal asymmetry [18,19].

Taken together, the genetic and functional evidence suggests a potential pathophysiological framework linking AIS-associated loci to tissue-level abnormalities and asymmetric spinal growth. Developmental susceptibility loci, including *LBX1* and *PAX1*, may influence somitic patterning, vertebral development, and neuromuscular organization through alterations in developmental gene regulation [7,10]. In parallel, *GPR126/ADGRG6* and *SOX6* are implicated in cartilage development and chondrocyte differentiation, suggesting that disturbances in these pathways could affect the maturation and mechanical properties of vertebral growth plates and intervertebral tissues [8,11]. *COL11A1* and *COL11A2* contribute to extracellular-matrix organization, particularly collagen fibril architecture, and may influence cartilage integrity and the biomechanical properties of developing spinal structures [13,14].

These molecular and cellular alterations may converge at the tissue level, potentially affecting vertebral growth plates, intervertebral discs, cartilage, and paraspinal musculature. During rapid adolescent growth, subtle differences in tissue maturation, structural integrity, or neuromuscular regulation could increase susceptibility to asymmetric loading and differential spinal growth. Such asymmetry may subsequently interact with mechanical forces, potentially contributing to the development or amplification of three-dimensional spinal deformity. This proposed sequence provides a conceptual link between genetic susceptibility and the characteristic structural manifestations of AIS.

Importantly, this framework represents a synthesis of genetic associations, experimental findings, and biomechanical hypotheses rather than a fully established causal pathway. The functional consequences of many human AIS-associated variants remain uncertain, and direct evidence connecting individual genetic variants to tissue-specific abnormalities and subsequent curve progression is limited. Further investigations integrating functional genomics, tissue-specific molecular analyses, longitudinal imaging, and biomechanical assessments are therefore required to validate these proposed relationships.

**Figure 1:**
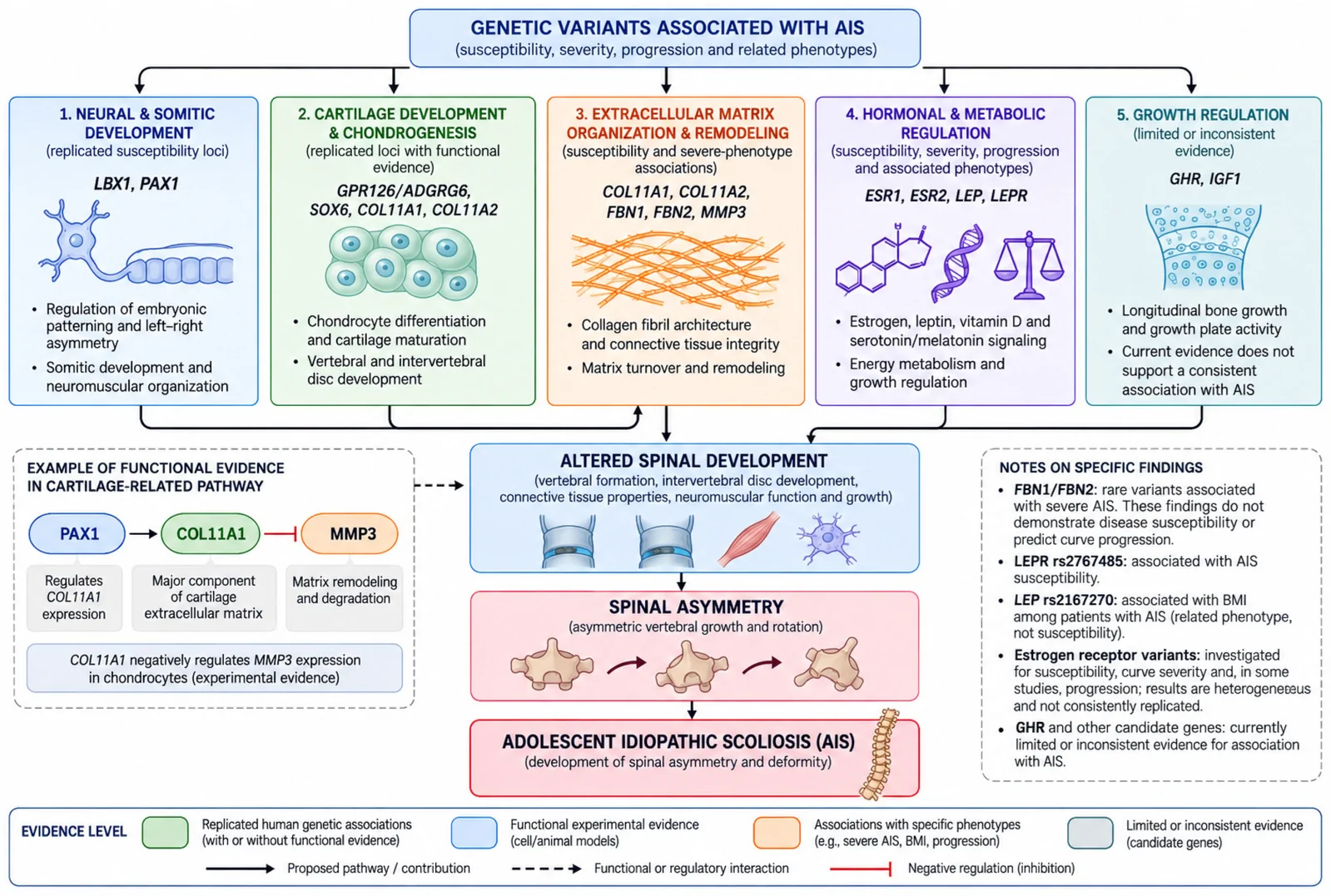
Genetic associations and proposed biological mechanisms underlying adolescent idiopathic scoliosis (AIS). The diagram distinguishes replicated human genetic associations, experimental functional evidence, and limited or hypothesis-generating associations. Functional investigations involving PAX1, GPR126/ADGRG6, and COL11A1 support selected developmental and cartilage-related mechanisms, although experimental gene disruption does not necessarily reproduce the effects of human AIS-associated variants. The inhibitory relationship between COL11A1 and MMP3 represents negative regulation observed in experimental chondrocyte models. Pathways supported by limited or inconsistent human association evidence are presented separately from established susceptibility loci. The proposed interactions do not establish individual clinical prediction of AIS onset or curve progression.

The principal biological pathways and genetic factors implicated in AIS are summarized in Table 2, while the proposed interactions between the major genetic pathways are illustrated in Figure 1.

## 5. Future Directions

Recent investigations have substantially expanded the understanding of AIS genetics beyond the initial candidate-gene and genome-wide association studies. Contemporary research incorporating multi-ancestry cohorts, whole-genome sequencing, functional genomic investigations, and phenotype-specific association analyses has provided new insights into the genetic heterogeneity of AIS. In particular, studies published between 2024 and 2026 have highlighted the contribution of *COL11A1*-related regulatory and extracellular-matrix mechanisms, coding and non-coding variation identified through whole-genome sequencing, and genetic loci associated specifically with curve severity. These advances underscore the importance of interpreting earlier susceptibility findings alongside newer molecular and functional evidence. Nevertheless, the translation of these discoveries into clinically validated prognostic tools remains an unresolved challenge [14,22,24].

### 5.1. Functional Validation of Susceptibility Loci

A major priority for future AIS genetic research is the functional characterization of susceptibility loci identified through genome-wide and rare-variant studies. Although multiple loci have reached statistical significance and several have been replicated across independent populations, the biological consequences of many associated variants remain incompletely understood. In particular, non-coding variants identified through GWAS may influence gene regulation rather than protein structure, making functional interpretation essential for distinguishing causal variants from linked genetic markers.

Studies involving *PAX1*, *GPR126/ADGRG6*, and *COL11A1* illustrate the value of integrating genetic association with functional investigation. Functional investigations have provided different levels of mechanistic support for selected AIS-associated loci. For *PAX1*, zebrafish enhancer assays demonstrated that AIS-associated regulatory variants can alter enhancer activity, supporting a potential effect on developmental gene regulation [10]. For *GPR126/ADGRG6*, experimental knockdown in zebrafish was associated with delayed spinal ossification, while conditional deletion in mouse osteochondroprogenitor cells produced postnatal scoliosis and abnormalities of intervertebral disc development [8,23]. For *COL11A1*, experiments involving *Pax1* knockout mice and genetically modified chondrocytes demonstrated altered collagen XI expression and regulation of Mmp3, providing evidence for a functional relationship between cartilage-associated pathways [14]. Importantly, experimental disruption of a gene does not necessarily demonstrate that the corresponding human AIS-associated variant produces the same biological effect.

Experimental models have demonstrated that AIS-associated variants may alter enhancer activity, gene expression, cartilage development, and extracellular-matrix regulation [8,10,14]. Future studies should extend these approaches through gene-expression analyses, genome editing, cellular models, organoid systems where appropriate, and animal models capable of reproducing relevant aspects of spinal development.

Particular attention should be given to the tissue and developmental stage in which susceptibility variants exert their effects. Because AIS becomes clinically apparent during adolescence but may originate from disturbances in earlier developmental programs, functional studies should investigate both embryonic and postnatal mechanisms. Clarifying the temporal and tissue-specific consequences of genetic variation will be essential for determining how susceptibility loci contribute to spinal asymmetry.

### 5.2. Multi-Omics Integration

Genetic variation alone is unlikely to explain the full biological complexity of AIS. Integration of genomic data with other molecular layers, including epigenomics, transcriptomics, proteomics, and metabolomics, may provide a more comprehensive understanding of how inherited susceptibility is translated into altered cellular and tissue function. Such approaches may also help explain why individuals carrying similar susceptibility variants can develop different clinical phenotypes.

Epigenetic mechanisms are particularly relevant to AIS because they provide a potential molecular interface between a stable genetic background and dynamic developmental or environmental influences. DNA methylation, histone modifications, chromatin organization, and non-coding RNAs can alter gene expression without changing the underlying DNA sequence and may therefore contribute to interindividual differences in disease expression [15,16,17]. In this context, hormonal status, metabolic conditions, mechanical loading, and other growth-related exposures could potentially influence transcriptional programs in genetically susceptible tissues. Dysregulated microRNA expression has also emerged as a potential component of AIS biology. Montemurro et al. [31] highlighted possible molecular overlap between AIS and 22q11.2 deletion syndrome, including dysregulation of miR-93 and miR-1306, and proposed altered microRNA regulation as a potential mechanism contributing to scoliosis-related molecular pathways. Although these observations remain exploratory and do not establish causality, they support further investigation of epigenetic regulation as a mechanism capable of linking inherited susceptibility with developmental and environmental influences. Multi-omics approaches may also help identify convergent molecular networks connecting apparently distinct susceptibility genes. Rather than examining *LBX1*, *PAX1*, *GPR126*, *COL11A1*, or other loci in isolation, integrated analyses could determine whether these genes ultimately affect shared developmental, cartilage, extracellular-matrix, or neuromuscular pathways. Such pathway-level interpretation may be more informative than focusing exclusively on individual variants.

Epigenetic regulation may represent an important interface between inherited susceptibility and developmental or environmental influences in AIS. In addition to DNA methylation and chromatin regulation, dysregulated microRNA expression has emerged as a potential mechanism capable of modifying gene-expression networks relevant to spinal development. Montemurro et al. [31] highlighted potential molecular overlap between AIS and 22q11.2 deletion syndrome, identifying miR-93 and miR-1306 as dysregulated in both conditions and proposing altered microRNA biogenesis and regulation as a possible shared mechanism. Although these observations do not establish a direct causal pathway for AIS, they support the broader concept that epigenetic mechanisms may modulate the phenotypic expression of an underlying genetic susceptibility.

### 5.3. Gene–Environment Interactions

The relationship between genetic susceptibility and developmental timing warrants particular attention in AIS. Although the deformity typically becomes clinically apparent during adolescence, several susceptibility genes implicated in AIS participate in biological processes that operate much earlier in development, including embryonic patterning, somite formation, neuromuscular development, chondrogenesis, and extracellular-matrix organization. Genetic variation affecting these processes may therefore establish subtle developmental differences in vertebral morphology, connective-tissue properties, neuromuscular organization, or spinal growth without necessarily producing an immediately detectable deformity.

Adolescence may subsequently represent a critical period during which these pre-existing biological differences become phenotypically expressed. Rapid longitudinal skeletal growth increases the mechanical and biological demands placed on the spine, while simultaneous changes in hormonal signaling, bone remodeling, body composition, and neuromuscular control may further modify tissue responses. Under this model, genetic susceptibility may establish a developmental substrate, whereas pubertal growth and associated biological or environmental influences may contribute to the emergence or amplification of spinal asymmetry. This temporal framework may help reconcile the involvement of genes acting during early development with the characteristic adolescent onset of clinically detectable scoliosis.

Importantly, this model should be regarded as a mechanistic framework rather than an established sequence of causal events. Longitudinal studies integrating genetic, epigenetic, developmental, hormonal, and biomechanical data from the preadolescent period through skeletal maturity will be required to determine when susceptibility-related biological differences emerge and which factors influence their progression toward clinically significant AIS.

### 5.4. Multi-Ethnic Genetic Studies

Another major limitation of current AIS genetic evidence is the restricted ancestral diversity of many study populations. Several influential GWASs were initially conducted in Japanese or Han Chinese cohorts, whereas subsequent studies demonstrated both replication and heterogeneity across populations. Large, multi-ethnic cohorts are therefore required to distinguish broadly shared susceptibility loci from population-specific genetic effects and to improve the generalizability of genetic findings.

Future studies should combine greater ancestral diversity with standardized and detailed phenotyping. Precise characterization of age at diagnosis, sex, curve pattern, Cobb angle, skeletal maturity, growth status, and relevant biological characteristics may help reduce phenotypic heterogeneity and improve genotype–phenotype analyses. Although the primary focus of susceptibility studies is disease risk rather than prognosis, detailed phenotyping may also help identify biologically distinct AIS subgroups with partially different genetic architectures.

International collaboration will likely be essential to achieve sufficiently large sample sizes for rare-variant analyses, ancestry-specific studies, and functional follow-up of newly identified loci. Harmonized phenotyping and shared genomic datasets could substantially improve statistical power and facilitate replication across independent populations.

### 5.5. From Genetic Association to Biological Understanding

The central challenge for future AIS genetics is therefore no longer simply the identification of additional associated loci, but the translation of genetic association into biological understanding. Increasing numbers of susceptibility variants will have limited explanatory value unless their functional consequences and interactions within relevant developmental pathways can be established.

Future research should prioritize the identification of causal variants, the definition of affected regulatory networks, and the characterization of their effects in biologically relevant tissues and developmental stages. Integrating common variants, rare variants, regulatory mechanisms, and multi-omics data within a unified biological framework may ultimately clarify how multiple modest genetic perturbations converge to create susceptibility to spinal deformity.

Such a transition from locus discovery toward mechanistic interpretation is essential for moving the field beyond cataloguing genetic associations and toward a more coherent understanding of AIS pathogenesis.

## 6. Conclusions

Current evidence supports AIS as a complex polygenic developmental disorder in which susceptibility arises from multiple levels of genetic variation rather than from a single causative gene or pathway. The progression from candidate-gene studies to genome-wide association analyses, rare-variant investigations, and functional genomics has identified increasingly robust susceptibility signals, including replicated loci such as *LBX1*, *GPR126/ADGRG6*, *PAX1*, and *SOX6*, together with emerging evidence implicating extracellular-matrix genes such as *COL11A1* and *COL11A2*. Functional investigations of *PAX1*, *GPR126*, and *COL11A1* further demonstrate the importance of moving beyond statistical association toward determining the biological consequences of genetic variation.

The principal contribution of this review is to integrate these different levels of genetic evidence within a pathway-based and developmental framework rather than considering AIS-associated genes as isolated findings. Across apparently distinct susceptibility loci, the available evidence converges on interconnected processes involving neural and somitic development, chondrogenesis, extracellular-matrix organization and remodeling, hormonal and metabolic regulation, and skeletal growth. This synthesis also highlights an important distinction between well-replicated genetic susceptibility loci and biological pathways for which the genetic or functional evidence remains less consistent. Hormonal, metabolic, environmental, and epigenetic influences may be particularly relevant as modifiers of the phenotypic expression of inherited susceptibility rather than as independent primary causes of AIS.

A further implication of this integrated framework is that the timing of genetic effects may differ from the timing of clinical disease expression. Susceptibility-related alterations in developmental or tissue-level processes may originate before adolescence, whereas rapid pubertal growth and associated hormonal, metabolic, biomechanical, and epigenetic influences may provide the biological context in which these differences become clinically apparent. This perspective may help bridge the apparent gap between genes involved in early developmental processes and the characteristic adolescent emergence of AIS.

Future research should therefore prioritize functional validation of established susceptibility loci, integration of genomic data with epigenomic, transcriptomic, proteomic, and metabolomic information, longitudinal investigation of gene–environment and gene–development interactions, and greater ancestral diversity in genetic studies. Such approaches will be necessary to move from lists of associated variants toward a mechanistic understanding of how inherited susceptibility is translated into spinal deformity and, ultimately, toward biologically informed approaches to risk stratification and personalized management in AIS.

## Figures and Tables

**Table 1:** Representative genetic studies illustrating the evolution of research on adolescent idiopathic scoliosis susceptibility.

Study	Population/Sample	Study Design	Gene/Locus	Variant(s)	Phenotype/Outcome	Main Finding/Effect	Replication/Evidence
Qiu et al., 2007 [18]	Chinese; 510 AIS/363 controls	Candidate-gene	*GHR*	rs6179, rs6176, rs6180, rs6184, and exon 3 deletion (d3-GHR)	Susceptibility; curve severity	No significant association	Negative association study; independent replication not established
Moon et al., 2013 [19]	Korean; 68 AIS cases/35 controls	Candidate-gene association	*IGF1*; *LAPTM4B*	rs5742612; rs2449539	Susceptibility; curve severity	Both variants were associated with AIS susceptibility and curve severity.	Single-center association; no independent replication
Araujo Junior et al., 2024 [20]	Brazilian girls; 189 AIS cases/240 controls	Case–control genetic association	*LEPR*; *LEP*	rs2767485; rs2167270	Susceptibility; BMI	LEPR rs2767485 T allele: OR 1.53 (95% CI 1.09–2.13); TC + TT: OR 1.84 (95% CI 1.69–2.01). LEP rs2167270 associated with lower BMI, not AIS susceptibility	No independent replication reported
Takahashi et al., 2011 [7]	Japanese females; 1376 AIS cases/11,297 controls	GWAS and replication	*LBX1 region*	rs11190870	Susceptibility	OR 1.56; *p* = 1.24 × 10^−19^	Internal replication; subsequently replicated in other populations
Kou et al., 2013 [8]	Japanese; 1819 AIS cases/25,939 controls	GWAS and functional analysis	*GPR126/ADGRG6*	rs6570507	Susceptibility	OR 1.28 (95% CI 1.18–1.38); *p* = 2.25 × 10^−10^	Replication in Han Chinese and European-ancestry populations; zebrafish knockdown demonstrated delayed spinal ossification
Zhu et al., 2015 [9]	Chinese girls; 4317 AIS cases/6016 controls	Four-stage GWAS	*AJAP1*; *PAX3/EPHA4*; *BCL2*; *LBX1-AS1*	rs241215; rs13398147; rs4940576; rs678741	Susceptibility	Combined *p* = 2.95 × 10^−9^; 7.59 × 10^−13^; 2.22 × 10^−12^; 9.68 × 10^−37^, respectively	Multistage genetic replication
Sharma et al., 2015 [10]	US multi-ethnic cohorts (including non-Hispanic White participants) and an independent Japanese replication cohort; 3102 individuals in the two-stage GWAS.	GWAS and enhancer assays	*PAX1 regulatory locus*	rs6137473 and associated enhancer variants	Female-specific susceptibility	Combined OR 1.30; *p* = 2.15 × 10^−10^	Independent genetic replication; zebrafish assays showed altered enhancer activity
Khanshour et al., 2018 [11]	Multi-ethnic discovery and replication cohorts; European and East Asian ancestry. Discovery: 3798 AIS cases/29,678 controls; replication: 4158 AIS cases/58,781 controls	GWAS meta-analysis	*SOX6*; *ABO*; *CDH13* and other loci	rs1455114 (SOX6); rs687621 (ABO); rs4513093 (CDH13)	Susceptibility	Genome-wide significant associations were identified at multiple susceptibility loci, including SOX6, ABO, and CDH13.	Multi-ethnic replication
Kou et al., 2019 [12]	Japanese; 79,211 individuals across three GWASs	GWAS meta-analysis	Multiple loci	Lead SNPs at 20 genome-wide significant loci	Susceptibility	14 novel susceptibility loci; associated lead variants explained approximately 4.6% of AIS phenotypic variance	Meta-analysis of three GWASs; cis-eQTL evidence for genes at seven novel loci
Haller et al., 2016 [13]	European ancestry; 391 severe AIS cases/843 controls; expanded validation	Exome sequencing; rare-variant burden analysis	*COL11A2* and other *ECM* genes	Rare nonsynonymous and splice-site variants	Severe AIS/susceptibility	ECM burden OR 1.7 (95% CI 1.4–2.0), *p* = 6 × 10^−9^; COL11A2 burden OR 3.8 (95% CI 2.6–7.2), *p* = 6 × 10^−9^	Targeted resequencing in a combined 919 AIS cases supported collagen-gene burden
Yu et al., 2024 [14]	Multi-ancestry cohorts; 10,519 AIS cases/93,238 controls	Matrisome-wide genetic association and functional investigation	*COL11A1*	rs3753841, c.4004C>T, p.Pro1335Leu; common missense variant	AIS susceptibility	Combined OR 1.118; *p* = 7.07 × 10^−11^	Multi-cohort genetic evidence; functional investigation of PAX1-related cartilage pathways
Xu et al., 2017 [21]	Chinese; 1952 AIS cases/2492 controls and 693 AIS cases/254 controls,	Genetic association and functional analysis	*BNC2*	rs10738445	Susceptibility; regulatory function	Association of rs10738445 with AIS susceptibility and altered BNC2 expression.	Genetic association and functional evidence supporting a regulatory role for BNC2.
Karner et al., 2015 [23]	Conditional knockout mice	Experimental gene deletion	*Gpr126/Adgrg6*	Conditional deletion in osteochondroprogenitor cells	Experimental scoliosis phenotype	Postnatal scoliosis, abnormal annulus fibrosus development and increased chondrocyte apoptosis	Direct experimental evidence; human association OR not applicable
Tuncay et al., 2025 [22]	119 affected individuals from 103 families	Whole-genome sequencing	Multiple genes, including *COL11A2*, *FBN1* and *RYR1*	Coding, non-coding and structural candidate variants	Genetic architecture/candidate variants	Pathogenic or protein-truncating variants in 15 probands; additional candidate variants in 69 patients	Family-based sequencing evidence; pathway enrichment, without a directly comparable case–control OR
Dai et al., 2026 [24]	Han Chinese females with AIS; discovery cohort: 620 patients; independent replication cohort: 1180 patients	Two-stage GWAS with independent replication	*GAK*; *KLF12*	rs2061846 (GAK); rs7330031 (KLF12)	Curve severity (severe vs. mild AIS)	OR 1.32 (1.11–1.57); OR 1.46 (1.16–1.85)	Both loci replicated in an independent cohort within the study

AIS, adolescent idiopathic scoliosis; GWAS, genome-wide association study; ECM, extracellular matrix; WGS, whole-genome sequencing; SNP, single-nucleotide polymorphism; OR, odds ratio; CI, confidence interval; BMI, body mass index; NA, not applicable; NR, not verified. Effect estimates refer to the specified genetic association or burden analysis and are not directly comparable across study designs. Abbreviations: AIS, adolescent idiopathic scoliosis; GWAS, genome-wide association study; ECM, extracellular matrix; OR, odds ratio; CI, confidence interval; BMI, body mass index; eQTL, expression quantitative trait locus.

**Table 2:** Major biological pathways and genetic factors implicated in adolescent idiopathic scoliosis.

Biological Pathway	Principal Genes	Proposed Biological Role	Relevance to AIS	References
Neural and somitic development	*LBX1*, *PAX1*	Regulation of embryonic patterning, somitic development, neuromuscular organization, and vertebral formation	Variants affecting developmental genes and their regulatory elements may predispose the growing spine to structural asymmetry. *PAX1* enhancer variants provide functional evidence that altered developmental gene regulation may contribute to AIS.	[7,10,14]
Cartilage development and chondrogenesis	*GPR126 (ADGRG6)*, *SOX6*, *COL11A1*, *COL11A2*	Regulation of chondrocyte differentiation, cartilage maturation, collagen organization, and vertebral development	Genetic and functional evidence suggests that impaired cartilage development and integrity may alter vertebral growth and increase susceptibility to asymmetric spinal deformation.	[8,11,13,14]
Extracellular matrix organization and remodeling	*COL11A1*, *COL11A2*, *FBN1*, *FBN2*, *MMP3*	Maintenance of collagen fibril architecture, connective-tissue integrity, elastic microfibrils, and extracellular-matrix turnover	Genetic and functional studies implicate extracellular matrix biology in AIS. *COL11A1* is associated with AIS susceptibility, whereas rare variants in *FBN1* and *FBN2* have been reported in severe AIS cohorts. These findings concern distinct clinical outcomes and do not establish prospective prediction of curve progression. Experimental evidence also supports a regulatory relationship involving *PAX1*, *COL11A1*, and *MMP3*.	[13,14,25]
Hormonal and metabolic regulation	*ESR1/ESR2*, *LEP/LEPR*, vitamin D signaling, serotonin–melatonin signaling	Estrogen signaling, leptin signaling, vitamin D metabolism, and serotonin/melatonin-related signaling	*LEPR* rs2767485 has been associated with AIS susceptibility, whereas *LEP* rs2167270 has been associated with BMI among patients with AIS. Estrogen receptor studies have investigated heterogeneous outcomes, including susceptibility, severity, and progression, with inconsistent replication. These findings do not establish clinically validated predictors of curve progression.	[20,26,27,28]
Growth regulation	*GHR*, *IGF1*	Regulation of longitudinal skeletal growth, growth-plate activity, and vertebral development	The growth hormone–*IGF1* axis regulates longitudinal skeletal growth and growth-plate activity. Current evidence does not support GHR as a major independent AIS susceptibility gene, whereas *IGF1* variation has been associated with AIS-related phenotypes in selected populations. Growth-related pathways may modify the phenotypic expression of genetic susceptibility during adolescence.	[18,19]

Abbreviations: AIS, adolescent idiopathic scoliosis; ECM, extracellular matrix; GHR, growth hormone receptor; IGF1, insulin-like growth factor 1.

## Data Availability

No new data were created or analyzed in this study. Data sharing is not applicable to this article.

## Abbreviations

ADGRG6	Adhesion G protein-coupled receptor G6
AIS	Adolescent idiopathic scoliosis
BMI	Body mass index
COL11A1	Collagen type XI alpha 1 chain
COL11A2	Collagen type XI alpha 2 chain
DNA	Deoxyribonucleic acid
ECM	Extracellular matrix
ESR1	Estrogen receptor 1
ESR2	Estrogen receptor 2
FBN1	Fibrillin 1
FBN2	Fibrillin 2
GHR	Growth hormone receptor
GPR126	G protein-coupled receptor 126
GWAS	Genome-wide association study
IGF1	Insulin-like growth factor 1
LBX1	Ladybird homeobox 1
LEP	Leptin
LEPR	Leptin receptor
MMP3	Matrix metallopeptidase 3
PAX1	Paired box 1
SNP	Single-nucleotide polymorphism
SOX6	SRY-box transcription factor 6
VDR	Vitamin D receptor

## References

[1] Cheng J.C., Castelein R.M., Chu W.C., Danielsson A.J., Dobbs M.B., Grivas T.B., Gurnett C.A., Luk K.D., Moreau A., Newton P.O. (2015). Adolescent idiopathic scoliosis. Nat. Rev. Dis. Primers.

[2] Herdea A., Stancu T.A., Ulici A., Lungu C.N., Dragomirescu M.C., Charkaoui A. (2022). Quality of Life Evaluation Using SRS-30 Score for Operated Children and Adolescent Idiopathic Scoliosis. Medicina.

[3] Popescu M.B., Marie H., Ulici A., Ionescu S.N., Dragomirescu M.C., Popescu C., Herdea A. (2026). Preoperative Halo Traction Versus Direct Posterior Fusion in Severe Adolescent Idiopathic Scoliosis: A Comparative Study. J. Clin. Med..

[4] Grauers A., Rahman I., Gerdhem P. (2012). Heritability of scoliosis in the Swedish Twin Registry. Eur. Spine J..

[5] Ward K., Ogilvie J., Argyle V., Nelson L., Meade M., Braun J., Chettier R. (2010). Polygenic inheritance of adolescent idiopathic scoliosis: A study of extended families in Utah. Am. J. Med. Genet A.

[6] Wise C.A., Gao X., Shoemaker S., Gordon D., Herring J.A. (2008). Understanding genetic factors in idiopathic scoliosis, a complex disease of childhood. Curr. Genom..

[7] Takahashi Y., Kou I., Takahashi A., A Johnson T., Kono K., Kawakami N., Uno K., Ito M., Minami S., Yanagida H. (2011). A genome-wide association study identifies common variants near LBX1 associated with adolescent idiopathic scoliosis. Nat. Genet..

[8] Kou I., Takahashi Y., A Johnson T., Takahashi A., Guo L., Dai J., Qiu X., Sharma S., Takimoto A., Ogura Y. (2013). Genetic variants in GPR126 are associated with adolescent idiopathic scoliosis. Nat. Genet..

[9] Zhu Z., Tang N.L.-S., Xu L., Qin X., Mao S., Song Y., Liu L., Li F., Liu P., Yi L. (2015). Genome-wide association study identifies new susceptibility loci for adolescent idiopathic scoliosis in Chinese girls. Nat. Commun..

[10] Sharma S., Gao X., Zhang D., Mauldin K., Kamiya N., Cornelia R., Wise C.A., Londono D., Gordon D., Eckalbar W.L. (2015). A PAX1 enhancer locus is associated with susceptibility to idiopathic scoliosis in females. Nat. Commun..

[11] Khanshour A.M., Kou I., Fan Y., Einarsdottir E., Makki N., Kidane Y.H., Kere J., Grauers A., A Johnson T., Paria N. (2018). Genome-wide meta-analysis and replication studies in multiple ethnicities identify novel adolescent idiopathic scoliosis susceptibility loci. Hum. Mol. Genet..

[12] Kou I., Otomo N., Takeda K., Momozawa Y., Lu H.-F., Kubo M., Kamatani Y., Ogura Y., Takahashi Y., Nakajima M. (2019). Genome-wide association study identifies 14 previously unreported susceptibility loci for adolescent idiopathic scoliosis in Japanese. Nat. Commun..

[13] Haller G., Alvarado D., Mccall K., Yang P., Cruchaga C., Harms M., Goate A., Willing M., Morcuende J.A., Baschal E. (2016). A polygenic burden of rare variants across extracellular matrix genes among individuals with adolescent idiopathic scoliosis. Hum. Mol. Genet..

[14] Yu H., Khanshour A.M., Ushiki A., Otomo N., Koike Y., Einarsdottir E., Fan Y., Antunes L., Kidane Y.H., Cornelia R. (2024). Association of genetic variation in COL11A1 with adolescent idiopathic scoliosis. eLife.

[15] Pérez-Machado G., Berenguer-Pascual E., Bovea-Marco M., Rubio-Belmar P.A., García-López E., Garzón M.J., Mena-Mollá S., Pallardó F.V., Bas T., Viña J.R. (2020). From genetics to epigenetics to unravel the etiology of adolescent idiopathic scoliosis. Bone.

[16] Faldini C., Manzetti M., Neri S., Barile F., Viroli G., Geraci G., Ursini F., Ruffilli A. (2022). Epigenetic and Genetic Factors Related to Curve Progression in Adolescent Idiopathic Scoliosis: A Systematic Scoping Review of the Current Literature. Int. J. Mol. Sci..

[17] Sun D., Ding Z., Hai Y., Cheng Y. (2023). Advances in epigenetic research of adolescent idiopathic scoliosis and congenital scoliosis. Front Genet..

[18] Qiu X.S., Tang N.L.S., Yeung H.Y., Qiu Y., Cheng J.C.Y. (2007). Genetic association study of growth hormone receptor and idiopathic scoliosis. Clin. Orthop. Relat. Res..

[19] Moon E.S., Kim H.S., Sharma V., Park J.O., Lee H.M., Moon S.H., Chong H.S. (2013). Analysis of single nucleotide polymorphism in adolescent idiopathic scoliosis in Korea: For personalized treatment. Yonsei Med. J..

[20] Junior A.E.P.A., de Azevedo G.B.L., Moliterno L.A.M., Tavares R.H., Cardoso J.V., de Souza G.R., Guimarães J.A.M., Defino H.L.A., Perini J.A. (2024). Association of polymorphism in leptin receptor gene with susceptibility of adolescent idiopathic scoliosis. Eur. Spine J..

[21] Xu L., Xia C., Qin X., Sun W., Tang N.L.S., Qiu Y., Cheng J.C.Y., Zhu Z. (2017). Genetic variant of BNC2 gene is functionally associated with adolescent idiopathic scoliosis in Chinese population. Mol. Genet Genom..

[22] Tuncay I.O., Lee E.K., Gustafson A., Lee Y., Jung D., Koh J.Y., Lee W., Lee S., Shazand K. (2025). Whole genome sequencing in adolescent idiopathic scoliosis cohort implicates multiple biological pathways. npj Genom. Med..

[23] Karner C.M., Long F., Solnica-Krezel L., Monk K.R., Gray R.S. (2015). Gpr126/Adgrg6 deletion in cartilage models idiopathic scoliosis and pectus excavatum in mice. Hum. Mol. Genet..

[24] Dai Z., Wu Z., Xu L., Feng Z., Qiu Y., Zhu Z. (2026). Genome-wide association study identifies GAK and KLF12 associated with curve severity of adolescent idiopathic scoliosis. PeerJ.

[25] Buchan J.G., Alvarado D.M., Haller G.E., Cruchaga C., Harms M.B., Zhang T., Willing M.C., Grange D.K., Braverman A.C., Miller N.H. (2014). Rare variants in FBN1 and FBN2 are associated with severe adolescent idiopathic scoliosis. Hum. Mol. Genet..

[26] Rao J., Qian S., Li X., Xu Y. (2024). Single nucleotide polymorphisms of estrogen receptors are risk factors for the progression of adolescent idiopathic scoliosis: A systematic review and meta-analyses. J. Orthop. Surg. Res..

[27] Dai J., Lv Z.T., Huang J.M., Cheng P., Fang H., Chen A.M. (2018). Association between polymorphisms in vitamin D receptor gene and adolescent idiopathic scoliosis: A meta-analysis. Eur. Spine J..

[28] Nelson L.M., Ward K., Ogilvie J.W. (2011). Genetic variants in melatonin synthesis and signaling pathway are not associated with adolescent idiopathic scoliosis. Spine.

[29] Ogura Y., Kou I., Takahashi Y., Takeda K., Minami S., Kawakami N., Uno K., Ito M., Yonezawa I., Kaito T. (2017). A functional variant in MIR4300HG, the host gene of microRNA MIR4300 is associated with progression of adolescent idiopathic scoliosis. Hum. Mol. Genet..

[30] Wang Y., Dai Z., Wu Z., Feng Z., Liu Z., Sun X., Xu L., Qiu Y., Zhu Z. (2021). Genetic variant of MIR4300HG is associated with progression of adolescent idiopathic scoliosis in a Chinese population. J. Orthop. Surg. Res..

[31] Montemurro N., Ricciardi L., Scerrati A., Ippolito G., Lofrese G., Trungu S., Stoccoro A. (2022). The Potential Role of Dysregulated miRNAs in Adolescent Idiopathic Scoliosis and 22q11.2 Deletion Syndrome. J. Pers. Med..

[32] Janusz P., Chmielewska M., Andrusiewicz M., Kotwicka M., Kotwicki T. (2021). Methylation of Estrogen Receptor 1 Gene in the Paraspinal Muscles of Girls with Idiopathic Scoliosis and Its Association with Disease Severity. Genes.

[33] Chmielewska M., Janusz P., Andrusiewicz M., Kotwicki T., Kotwicka M. (2020). Methylation of estrogen receptor 2 (ESR2) in deep paravertebral muscles and its association with idiopathic scoliosis. Sci. Rep..

[34] Hassan A., Parent S., Mathieu H., Zaouter C., Molidperee S., Bagu E.T., Barchi S., Villemure I., A Patten S., Moldovan F. (2019). Adolescent idiopathic scoliosis associated POC5 mutation impairs cell cycle, cilia length and centrosome protein interactions. PLoS ONE.

[35] Grimes D.T., Boswell C.W., Morante N.F.C., Henkelman R.M., Burdine R.D., Ciruna B. (2016). Zebrafish models of idiopathic scoliosis link cerebrospinal fluid flow defects to spine curvature. Science.

[36] Lu H., Shagirova A., Goggi J.L., Yeo H.L., Roy S. (2020). Reissner fibre-induced urotensin signalling from cerebrospinal fluid-contacting neurons prevents scoliosis of the vertebrate spine. Biol. Open.

